# Tracking Animal-Dispersed Seedlings Using ^15^N Xylem Injection Method

**DOI:** 10.3389/fpls.2021.582530

**Published:** 2021-04-30

**Authors:** Minghui Wang, Sijie Yi, Mengyao Ju, Xianfeng Yi

**Affiliations:** ^1^College of Life Sciences, Qufu Normal University, Qufu, China; ^2^College of Life and Environmental Sciences, University of Exeter, Exeter, United Kingdom

**Keywords:** seed dispersal, isotopic clue, ^15^N labeling, xylem injection, seedling recruitment

## Abstract

Although various seed-marking methods have been developed for seed dispersal, it remains difficult to track the actual patterns of seed dispersal and seedling recruitment. Thus, new labeling methods that accurately track seedling establishment along with seed movement would help us better understand seed dispersal. Here, we developed a new nondestructive method using ^15^N xylem injection to track seed dispersal and seedling recruitment based on the enriched isotopic signals in the mature seeds. Our results first showed that xylem injection of ^15^N successfully enriched ^15^N both in the acorns and seedlings of *Quercus variabilis*. By marking acorns and seedlings with ^15^N stable isotopes, we successfully tracked seedlings established from acorns dispersed by seed-eating animals in the field. Our xylem ^15^N injection caused little alteration to seeds and showed no significant effects on seed selection by seed-eating animals as well as seed germination and seedling establishment, verifying the validity of the ^15^N xylem injection method to track seedling establishment. Our xylem ^15^N injection method is expected to be a powerful tool for tracking seed dispersal and seedling recruitment mediated by seed-eating animals in seed dispersal ecology.

## Introduction

Understanding the factors affecting the composition and abundance of tree regeneration is critical to the natural succession of various forest ecosystems (Lichti et al., 2014; Montoro Girona et al., 2018). Natural regeneration of natural forests is mainly dependent on plant–animal interactions (Steele et al., 2018), in which seed dispersal mediated by seed-eating animals is vital in successional patterns of structure and composition of forests (Yang et al., 2020).

Seed dispersal, seed movement away from mother trees, shows prominent effects on the genetic structures, population dynamics, and community ecology of all ecosystems (Levine and Murrell, 2003; Yi et al., 2011). Seed dispersers play a critical role in dispersing seeds of many plant species in various ecosystems (Moore et al., 2007; Yi et al., 2011). However, to accurately estimate success of dispersed seedlings remains a challenging field research problem, because it is often difficult to determine seed movements mediated by seed-eating animals (Davis et al., 2011; Muñoz and Bonal, 2011; Hirsch et al., 2012a,b). These difficulties are largely attributed to methodological limitations to identify where the seeds germinate after seed dispersal (Galetti et al., 2010; Yi and Wang, 2015), which hampers better understanding of the ecological and evolutionary consequence of seed dispersal. Therefore, a better assessment of seed dispersal impacts on community ecology and population dynamics is highly dependent on developing new approaches to reveal where the seeds are deposited by seed-eating animals and whether they successfully escape predation and establish in the suitable conditions (Hirsch et al., 2012b; Yi et al., 2014).

During the past several decades, several methods have been developed for tracking seed dispersal. Inserting metal or magnets into large seeds and detecting with metal detectors appear to be a pioneering attempt (Alverson and Díaz, 1989; Den Ouden et al., 2005; Moore et al., 2007; Lichti et al., 2014). Later, some researchers attempted to dye seeds with fluorescent microspheres or powders for tracking (Lemke et al., 2010; Wróbel and Zwolak, 2013). Labeling seeds with threads or tags has been widely accepted and used in various studies (Yi and Zhang, 2008; Deng et al., 2020; Yang et al., 2020). In recent years, passive integrated transponders (PITs) and radio transmitters were used in seed dispersal ecology despite their high technical requirements (Steele et al., 2011; Hirsch et al., 2012b; Suselbeek et al., 2013). Most recently, Gu et al. (2017) and White et al. (2017) investigated the relationships between rodents and seeds at the individual level combining infrared radiation camera tracking and seed tagging. Although these methods have been used in many ecosystems for tracking seed dispersal, the main shortcoming is the influence of seed alteration on either seed choice by animals or the following seedling establishment (Levey and Sargent, 2000; Godoy and Jordano, 2001; Tewksbury et al., 2002; Hirsch et al., 2012a,b; Yi and Wang, 2015; Niu et al., 2020). Xiao and Zhang (2003) have well compared a majority of tracking methods, pointing out their advantages and limitations and potential implications in seed dispersal ecology.

Stable isotope labeling has been regarded as a powerful quantitative technique widely used in ecological research (Schmidt and Scrimgeour, 2001; Putz et al., 2011). Carlo and Norris (2012) first showed that petal surfaces can effectively capture and quickly allocate nitrogen ions into ovaries and developing seeds. Carlo et al. (2009) for the first time tracked seed distribution of small herbaceous plants (*Solanum americanum* and *Capsicum annuum*) based on labeling seeds or fruits by foliar-spraying with a solution of ^15^N-urea during the flowering stage. Later, Castellano and Gorchov (2013) and Forster and Herrmann (2014) verified the feasibility of foliar application of ^15^N-labeled urea to enrich ^15^N in seeds and seedlings of shrubby *Lonicera maackii* and herbaceous *Eupatorium glaucescens* and *Sericocarpus tortifolius*. Although these methods cause little alteration to seeds, they are highly limited to plants small enough to adequately and uniformly be covered with the urea-^15^N spray. Therefore, it is not broadly applicable for woody plants with high trunks and large canopies. Recently, Yi et al. (2014) and Yi and Wang (2015) further developed the stable isotope technique to track seed dispersal of tree species by soaking seeds with ^15^N-urea solutions. This approach is highly efficient for tracking seedling establishment in the field based on the ^15^N isotopic clues in seeds and seedlings. However, directly soaking seeds with ^15^N isotopic solution may alter seed palatability and odor and then render feeding discrimination of seed-eating animals. Therefore, developing a simple and reliable seed-tracking method is highly needed to facilitate better understanding of seed dispersal ecology (Bologna et al., 2017).

It has been evidenced that biomass enriched in ^15^N can be produced by direct injections of ^15^N-labeled fertilizers into the plant vascular system (Swanston and Myrold, 1998; Proe et al., 2000; Nair et al., 2014). Horwath et al. (1992) first utilized an injection technique to apply ^15^N-enriched compounds into plants via a purpose-drilled hole reaching the cambium and xylems where the solution was taken up via a Venturi effect (Nair et al., 2014). This procedure consists of a reservoir of injection substrate introduced into the tree trunks either passively (Proe et al., 2000) or under pressure (Horwath et al., 1992; Swanston and Myrold, 1998). Although this method has been used to trace the fate of injected elements, to our knowledge, it has never been used with the primary purpose of creating ^15^N-labeled propagules to track seed dispersal and seedling establishment. Here, we developed a novel approach to overcome previous methodological limitations by tracking seed dispersal and seedling recruitment relying on ^15^N enrichment in seeds via xylem injections. In this study, ^15^NH_4_Cl and K^15^NO_3_ were used for the injections because both of their constituent ions are transported in the xylem stream (Marschner and Marschner, 2012). We expected that the ^15^NH_4_Cl and K^15^NO_3_ will be transported along the xylems of Chinese cork oak *Quercus variabilis* and finally incorporated into their acorns, generating ^15^N-enriched seeds. Our aim was to develop a new method to track seed dispersal using isotopic clues injected via xylems to seeds. We specifically addressed the following questions: (1) Can xylem injection of ^15^NH_4_Cl and K^15^NO_3_ produce ^15^N-enriched seeds and seedlings? (2) Given that the previous tracking methods usually show effects on seed handling by animals, we would like to answer whether xylem injection of ^15^NH_4_Cl and K^15^NO_3_ influences seed choice of seed-eating animals. (3) Existing literature has shown that tracking methods cause negative impacts on or even mortality to seeds and seedlings, and we also wanted to know if xylem injection of ^15^NH_4_Cl and K^15^NO_3_ influences seed germination and seedling establishment. (4) Can xylem injection of ^15^NH_4_Cl and K^15^NO_3_ be a reliable new approach to track seedling recruitment in the field?

## Materials and Methods

### Injection of ^15^N Isotope Into the Xylems

In early July 2018 when the tiny fruits were setting, fruiting individuals of *Q. variabilis* were selected for ^15^N injection in a primary forest stand in Sichuan provinces in southwest China (Supplementary Figure 1, also see detailed information in Yang et al., 2020). We selected *Q. variabilis* trees with DBH (diameter at breast height) ranging from 30 to 40 cm > 100 m apart each other scattered in a sub-tropical forest for xylem ^15^N injection. Considering the limited area of the primary forest, we selected seven focal trees to avoid potential interference between their seedlings originated in the next year. The passive injection system was used to inject 60 mmol/L ^15^NH_4_Cl and K^15^NO_3_ (enriched to 10 atom% ^15^N, dissolved in 1,500 ml of distilled water) into the xylem with the aid of a 1,500-ml plastic infusion bag (Guoguang, Sichuan, China), which was inversely mounted on the trunk 2 m from the ground (Proe et al., 2000; Supplementary Figure 2). First, we removed an area (less than 15 cm^2^) of bark on the two opposite sides of the stem at the breast height. Then, two small opposite holes measuring 4 mm in diameter and 4 cm in depth were drilled using an electric hand drill with an auger bit (4 mm in diameter). Once drilled, two plastic tubes of 4 mm diameter were led through the bag top into the two holes on each tree stem. Immediately, we used a commercially available waterproof silicone sealant to coat the sides of holes to avoid potential cavitation (see Nair et al., 2014). The concentration of 60 mmol/L was intentionally selected because our previous attempts indicate that 40 mmol/L is not high enough to obtain ^15^N enriched seeds comparable to natural ones (Supplementary Figure 3). We checked every day to ensure all trees to successfully uptake ^15^N solution without obvious evidence of leaks. The solutions were taken up by all trees within 10 days. Once the solution was completely taken up by a given tree, the holes were refilled with silicon sealant.

### Measurement of ^15^N Isotopes in Acorns

In late September 2018 when fruits of *Q. variabilis* were ripened, we collected acorns under the canopy of each tree. To see if injection of ^15^NH_4_Cl and K^15^NO_3_ through xylem resulted in ^15^N enrichment in acorns, 30 acorns were randomly selected from each tree. For comparison, acorns from three to five control trees without ^15^N injection were collected around each focal tree in the same way for isotope measurement.

### Measurement of Nutrition in Acorns

Acorns were air-dried to constant weight in an oven at 70°C for 48 h to test if ^15^N injection significantly changed nutrition level of acorns. Crude protein content was determined by Kjeldahl method with digestion of sulfuric acid–hydrogen peroxide (Wang et al., 2014). Soxhlet extraction was used for determination of crude fat content (Gu et al., 2009). Total starch was determined by acidolysis-DNS (Lu and Li, 2002). The chlorine content was determined using silver nitrate titration method (Maines and Bruch, 2012). All samples were sent to the Cavenix Test Technology Co., Ltd (Nanjing, China) for analyses.

### Measurement of ^15^N Isotopes in Seedlings

To test if ^15^N injection showed any positive or negative effects on seed germination and seedling establishment, we randomly selected 50 ^15^N-enriched acorns from ^15^N enriched *Q. variabilis* trees for germination experiments. For comparison, 50 acorns were selected from a composite sample of control *Q. variabilis* trees, respectively. Acorns were individually planted 1 cm deep into the plastic flower pots (diameter × height: 8 cm × 15 cm) containing fine sand free of nutrition. To compare germination rates, 50 pots were evenly assigned into 10 groups both for the control and ^15^N-enriched acorns. Plant containers were kept at room temperature under 600–800 μmol⋅m^–2^⋅s^–1^ radiation of fluorescent lamps, regularly watered, and randomly arranged in space. After 60 days’ cultivation, seed germination and seedling dry mass were measured for each tree species to see if xylem injection of ^15^N affects seed germination and seedling establishment. To see if xylem injection of ^15^NH_4_Cl and K^15^NO_3_ successfully produced ^15^N-enriched seedlings, one fully developed leaf, representing a mature leaf at the age of 60 days, was collected from each seedling. Previous study has shown that the newly assimilated N and remobilized N from reserves have rather different ^15^N signatures for a seedling (Yang et al., 2019; Cui et al., 2020). In addition, ^15^N will be diluted in old leaves (Yi and Wang, 2015); we therefore selected mature leaves to create sub-samples for both the ^15^N-enriched and control seedlings for isotope measurements, as N assimilation from soil and N remobilization from reserves are expected to reach a state of balance (Yi and Ju, 2020).

### Tracking Seedling Recruitment Using Isotopic Signals in the Field

To investigate whether xylem injection of ^15^N was reliable for tracking seed dispersal kernel and seedling establishment, we conducted area-constrained searches defined by a 30-m radius to locate 1-year seedlings of *Q. variabilis* around the seven injected trees in the next spring. Locating seedlings involved a systematic, comprehensive search of eight radial belts (2 m wide, 30 m long) around each focal tree. Distance between each seedling to the nearest focal tree was measured. Then, like the indoor procedures, one fully developed leaf was sampled from each seedling for later identification by using ^15^N isotope analysis. We collected leaves from 811 seedlings for ^15^N isotope analysis.

### Stable Isotope Analysis

All samples for isotope analyses were air-dried to constant weight in an oven at 70°C for 48 h. Then, they were ground finely and placed in an isotope ratio spectrometer for isotopic analysis using elemental analyzer/continuous flow isotope ratio mass spectrometry following the method of Yi et al. (2014). Samples were analyzed for stable nitrogen isotope abundance and nitrogen concentration at the Institute of Applied Ecology, Chinese Academy of Sciences (Shenyang, China). Interface between element-analysis meter and spectrometer was Flash EA1112 HT (Thermo Finnigan, United States). Operation condition: oxidizing furnace temperature was 900°C, reducing furnace was 680°C, and pillar temperature was 40°C. The resulting N_2_ was purified in a vacuum line and injected in a Finnigan MAT 235 (dual) spectrometer (Thermo Fisher Scientific, Inc., United States) fitted with double inlet and collector systems. The results are expressed in δ^15^N relative to the standards in the conventional δ per mil notation with a standard deviation of 0.2 per mil:

δ^15^N = [(^15^N/^14^N) _*sample*_/(^15^N/^14^N) _*standard*_ − 1] × 1000

Where ^15^N/^14^N are the isotopic ratios of sample (seeds or leaves) and standard (atmospheric nitrogen).

### Seed Selection and Pilferage by Seed-Caching Animals in Enclosures

Enrichment of ^15^N may change seed nutrition and seed odor, which may also influence seed handling (e.g., eating, caching and pilfering) by seed-eating animals. To test whether seed-eating animals selectively consumed and cached the control and ^15^N-enriched seeds, we provided 20 paired control and ^15^N enriched acorns of *Q. variabilis* to each individual of scatter-hoarding rodent Edward’s long-tailed rat (*Leopoldamys edwardsi*) caged in the separate enclosures (10 × 10 × 1.5 m) supplied with bedding materials, nests, and drinking water. Five females and five males were used to see if there was difference in acorn preference between genders. The ground of each enclosure was covered with bricks to create an 8 × 8 grid of 64 evenly spaced shallow pits (length × width × depth: 24 cm × 12 cm × 6 cm) each separated by 1 m. The shallow pits were filled with fine sand to allow seed burial by *L. edwardsi*. A seed station (0.5 m^2^) was established at the center of each enclosure for presentation of acorns. The ^15^N-enriched and control acorns of *Q. variabilis* were marked with different colors for easy identification. Ten hours after seed placement and animal introduction, we carefully searched the enclosures to record the seed fates: intact *in situ* (IIS), eaten *in situ* (EIS), eaten after removal (EAR), intact after removal (IAR), scatter-hoarded (SH), and LH (larder-hoarded). Seed fate of each category was compared to see if *L. edwardsi* showed preference for the control or ^15^N-enriched acorns of *Q. variabilis*.

To test if xylem injection of N isotope affects cache pilferage by scatter-hoarding animals, we established 32 artificial caches containing 16 paired control and ^15^N-enriched acorns in each enclosure and allowed individual animal to pilfer freely for 10 h. In total, 10 *L. edwardsi* were tested individually in the enclosures. In this study, we used *L. edwardsi* for testing because this species has been regarded as the predominant scatter-hoarding rodents in the study area (Xiao et al., 2008; Zhang et al., 2008), and scatter-hoarding rodents are better pilferers than larder-hoarders (Wang et al., 2018). The remaining rodent species (*Apodemus draco*, *A. chevrieri*, *Niviventer confucianus*, and *N. fulvescens*) are predominant larder-hoarders and may contribute little to seed dispersal and seedling establishment (Chang and Zhang, 2011). We did not conduct paired-seed selection experiments in the field because it was impossible to exclude the background effects of abundant natural acorns and probably the ecological role of an avian seed disperser *Garrulus glandarius* in the study area.

### Data Analysis

We conducted all statistical analyses using SPSS 16.0. An independent *t*-test was used to test the effects of xylem ^15^N injection on δ^15^N values in acorns and seedlings of *Q. variabilis*. Differences in acorn nutrients (protein, fat, and starch) and minerals (Cl and N) between the control and ^15^N-enriched acorns were tested using independent *t*-tests. The same procedure was used to test if xylem injection of ^15^N affected seed germination and seedling performance. We used paired samples *t*-test to test if xylem injection of ^15^N affected seed selection and cache pilferage by seed-eating animals in the artificial enclosures. In this study, seedlings with δ^15^N values 10 order of magnitudes higher than those of the control seedlings were considered from the ^15^N-enriched seeds in the field (Yi and Wang, 2015).

## Results

### ^15^N Enrichment in Seeds and Seedlings

**FIGURE 1:**
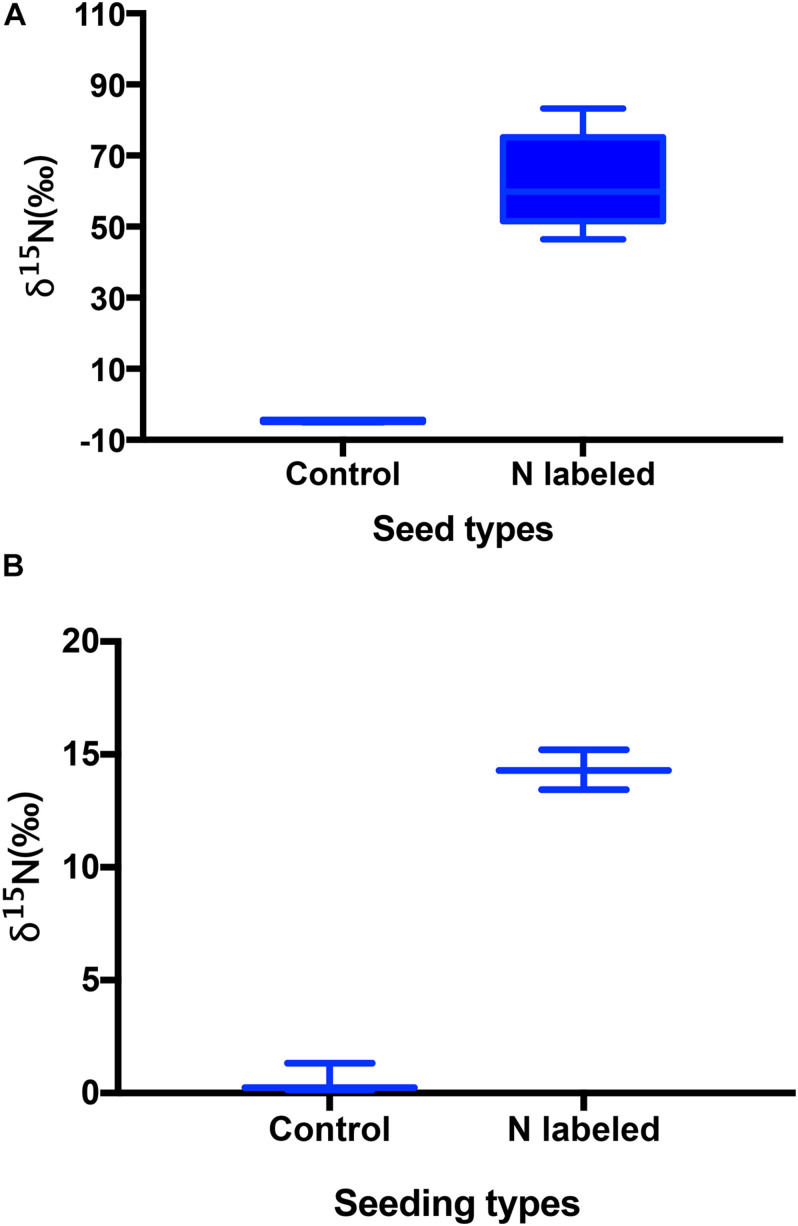
Effects of xylem injection of ^15^NH_4_Cl and K^15^NO_3_ on ^15^N enrichment of acorns **(A)** and seedlings **(B)**. Box plots show the median, upper (25%), and lower quartile (25%) of data and whiskers indicate the maximum and minimum values, with the exception of outliers, and the same below.

**FIGURE 2:**
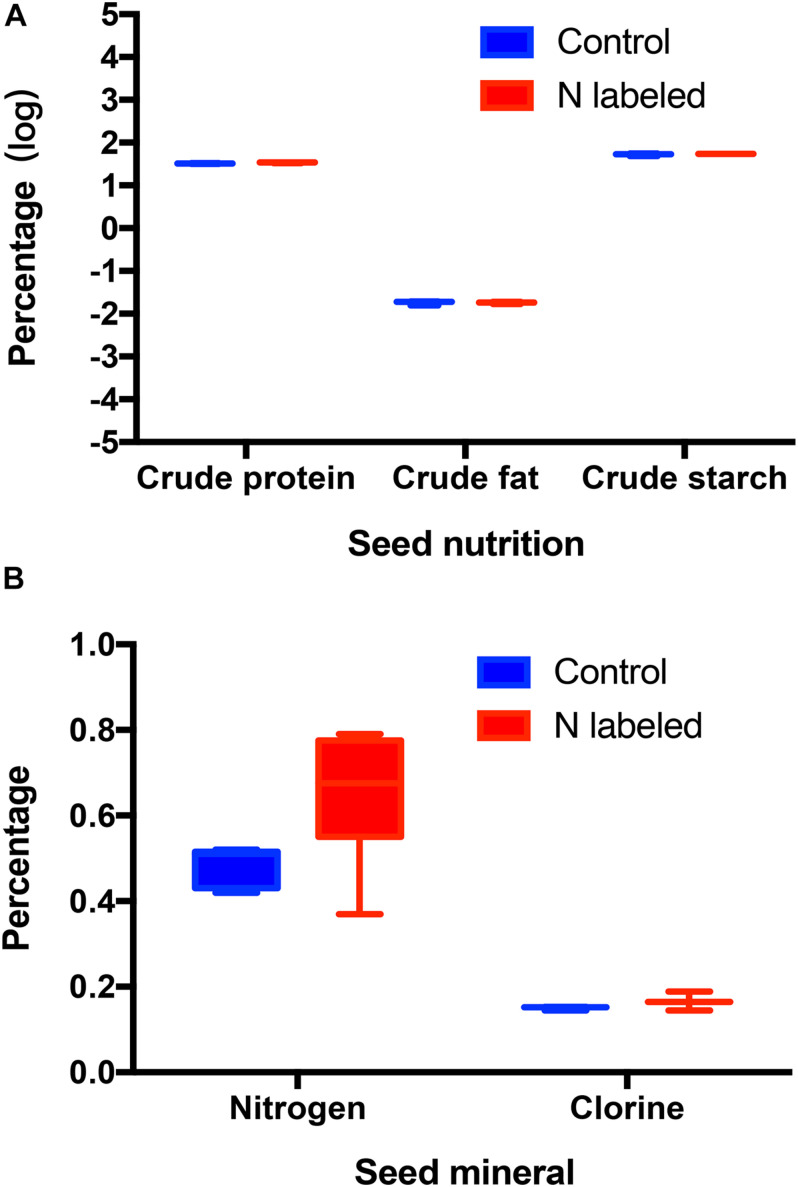
Effects of xylem injection of ^15^NH_4_Cl and K^15^NO_3_ on acorn nutrition **(A)** and minerals **(B)**.

Xylem injection using ^15^NH_4_Cl and K^15^NO_3_ caused significant ^15^N enrichment in seeds of *Q. variabilis* (*t* = −14.312, *df* = 9, *P* < 0.001) (Figure 1A). Correspondingly, ^15^N was observed significantly enriched in seedlings of *Q. variabilis* (*t* = −21.549, *df* = 4, *P* < 0.001) (Figure 1B). However, crude fat, starch, and chlorine in *Q. variabilis* acorns were not significantly affected by xylem ^15^N injection (*t* = 0.010, *df* = 4, *P* = 0.992; *t* = −0.961, *df* = 4, *P* = 0.391; *t* = −1.217, *df* = 4, *P* = 0.290), while the content of crude protein and N were slightly increased by xylem ^15^N injection (*t* = −2.803, *df* = 4, *P* = 0.049; *t* = −2.332, *df* = 9, *P* = 0.045) (Figures 2A,B).

### Effects of Xylem ^15^N Injection on Seed Germination and Seedling Establishment

**FIGURE 3:**
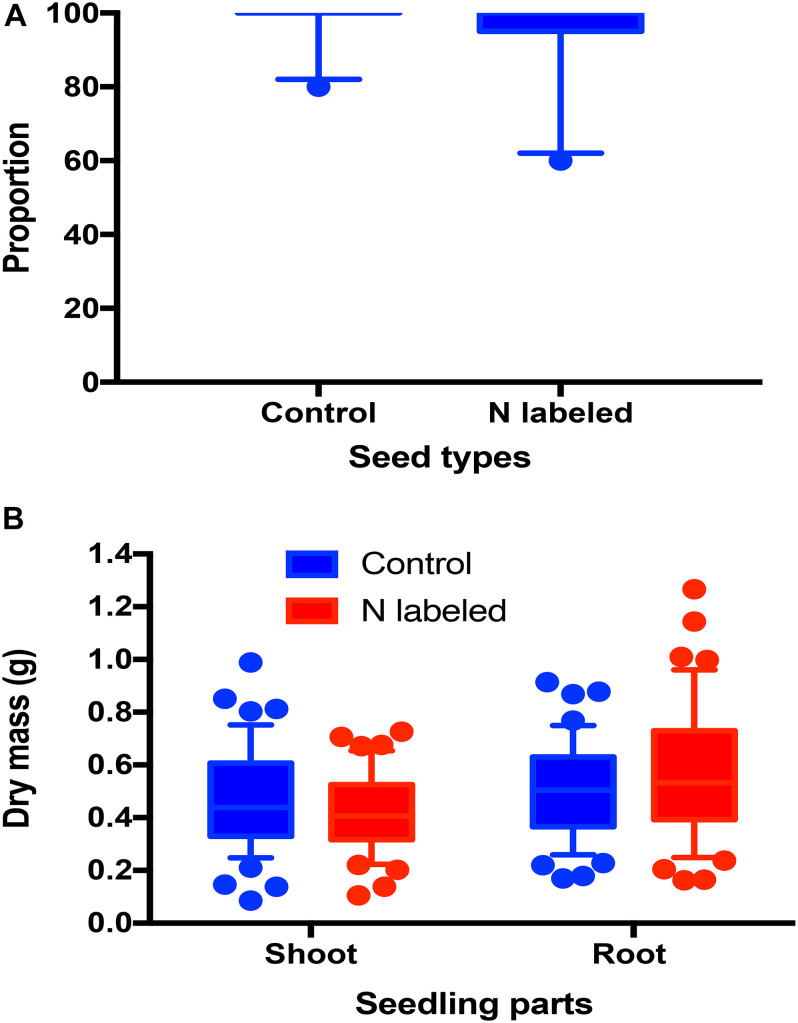
Effects of xylem injection of ^15^NH_4_Cl and K^15^NO_3_ on acorn germination rate **(A)** and shoot and root dry masses **(B)**.

Xylem ^15^N injection had no significant effect on seed germination rates of *Q. variabilis* (*t* = 0.849, *df* = 18, *P* = 0.407) (Figure 3A). Moreover, we detected no significant effects of xylem ^15^N injection on shoot and root dry mass of *Q. variabilis* seedlings (*t* = −1.474, *df* = 1, *P* = 0.114 *t* = 1.594, *df* = 87, *P* = 0.114; Figure 3B).

### Effects of Xylem ^15^N Injection on Seed Selection by *L. edwardsi*

**FIGURE 4:**
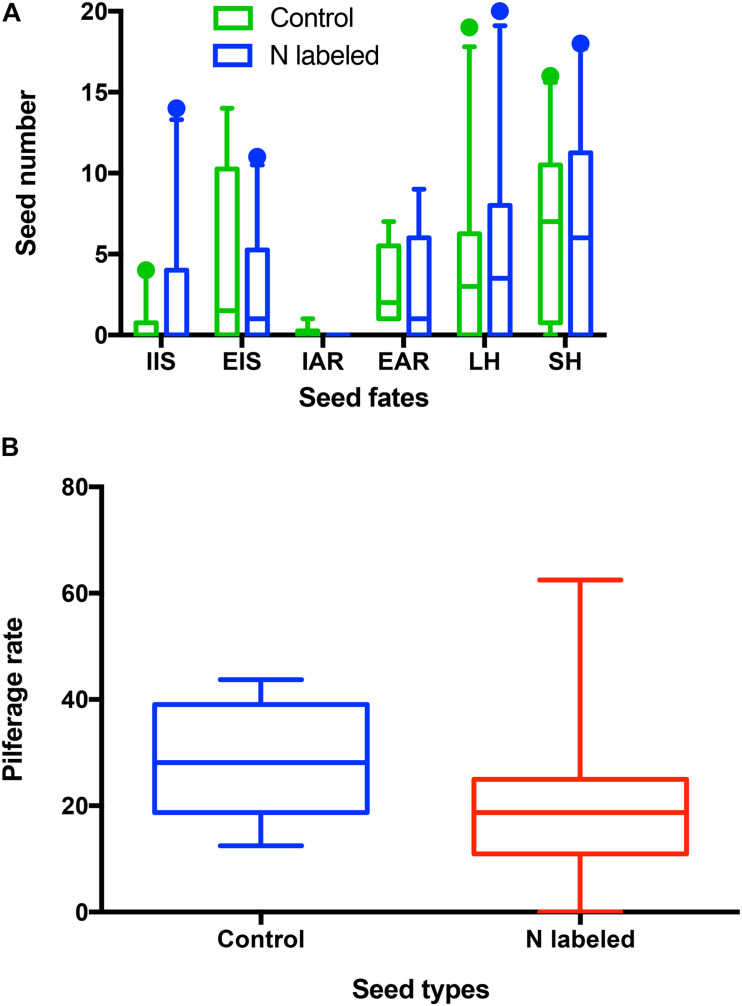
Effects of xylem injection of ^15^NH_4_Cl and K^15^NO_3_ on seed fates **(A)** and pilferage rate **(B)** of *Quercus variabilis* manipulated by *Leopoldamys edwardsi* in the enclosures. IIS, EIS, EAR, IAR, and SH stand for seeds intact *in situ*, eaten *in situ*, eaten after removal, intact after removal, and scatter-hoarded, respectively.

Overall, xylem ^15^N injection did not affect seed selection by *L. edwardsi*. In detail, acorns remaining intact *in situ* (IIS) were not different between the control and ^15^N-enriched groups after removed by *L. edwardsi* (*t* = −1.747, *df* = 9, *P* = 0.115) (Figure 4A). Similarly, no significant differences were found in acorns eaten *in situ* (EIS) (*t* = 2.023, *df* = 9, *P* = 0.074) and eaten after removal (EAR) (*t* = 1.060, *df* = 9, *P* = 0.317) (Figure 4A). Moreover, xylem ^15^N injection did not affect both scatter-hoarding (SH) and larder-hoarding by *L. edwardsi* (*t* = 1.300, *df* = 9, *P* = 0.226; *t* = −0.275, *df* = 9, *P* = 0.790) (Figure 4A). Xylem ^15^N injection showed no effect on cache pilferage by *L. edwardsi* (*t* = 1.450, *df* = 9, *P* = 0.181) (Figure 4B). Neither male nor female of *L. edwardsi* showed any preference to scatter-hoard the paired acorns (*t* = −0.250, *df* = 4, *P* = 0.815; *t* = −0.152, *df* = 4, *P* = 0.887). The same patterns were found in acorns eaten in situ, eaten after removal, larder-hoarded, and cache pilfering (all *P* > 0.05).

### Seedling Recruitment Based on Isotopic Signals in Seeds

**FIGURE 5:**
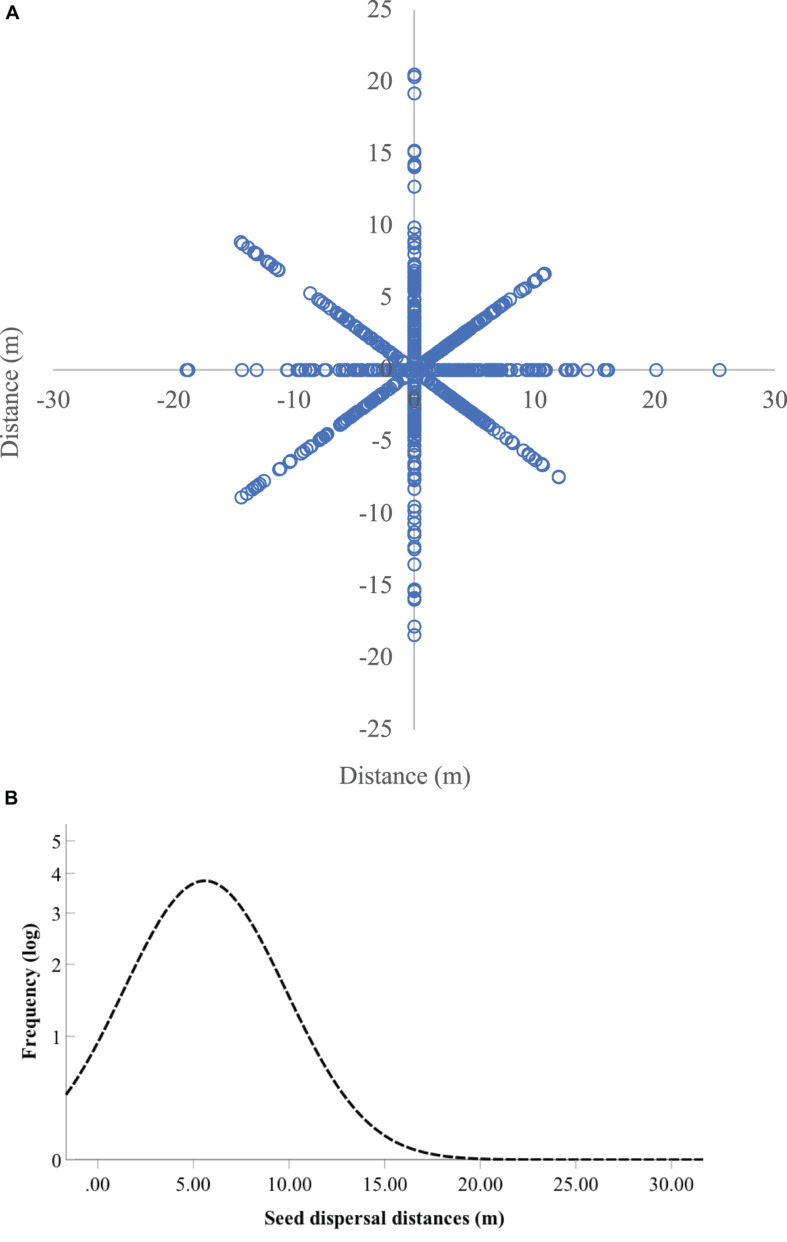
The spatial distribution pattern **(A)** and frequency distribution curve of observed dispersal distances **(B)** of *Quercus variabilis* seedlings in the field. In total, 811 seedlings were surveyed, in which 234 seedlings showed δ^15^N values higher than the natural ones.

In spring 2019, we totally located 811 1-year seedlings of *Q. variabilis* around the seven focal trees through area-constrained searches by three people for 10 days (Figure 5A). Based on the isotope analyses, 234 seedlings of *Q. variabilis* showed δ^15^N values higher than 15‰ (average 23.01‰) and were regarded to be originated from the seven focal parent trees that received ^15^N xylem injection (Supplementary Figure 4). The remaining seedlings (577, 71.1%), however, exhibited extreme low δ^15^N values (average 1.45‰) and were considered as natural seedlings originated from the untreated *Q. variabilis* trees. The frequency distribution curve of *Q. variabilis* seedlings near the parent trees appeared right-skewed (Figure 5B), generating an average distance between the ^15^N-enriched seedlings and the corresponding trees of *Q. variabilis* estimated at 5.6 m.

## Discussion

Our experiments clearly showed that xylem ^15^N injection of 60 mmol/L ^15^NH_4_Cl and K^15^NO_3_ successfully resulted in^ 15^N enrichment in acorns of *Q. variabilis*. Most importantly, ^15^N enrichment was also successfully detected in the 1-year seedlings of *Q. variabilis* receiving xylem ^15^N injection. The apparent differences in δ^15^N values between the control and ^15^N-enriched seedlings helped us easily track seedling recruitment in the field (Supplementary Figure 4). Compared to the δ^15^N values of the natural seedlings, we successfully tracked seedlings produced by the ^15^N-enriched seeds of *Q. variabilis* in the field, verifying the reliability of xylem injection of ^15^NH_4_Cl and K^15^NO_3_ to mark seeds for tracking seedling recruitment. The shape of the dispersal kernel, i.e., the probability distribution of dispersal distances, has great influence on the ultimate distribution of genetic diversity within populations. However, in contrast to the highly left-skewed (fat tail) distributions reported for many empirical studies (Goto et al., 2006; Morales and Carlo, 2006; Hardy, 2009), we find that the dispersal distribution in *Q. variabilis* near the parent trees was thin-tailed and right-skewed (Figure 5B). The thin-tailed and right-skewed shape of the *Q. variabilis* seedling distribution does imply that long-distance dispersal is rare in this species (Barlow et al., 2013). In many species, seedling mortality decreases with increasing distance from the mother plants (Peters, 2003). However, acorns of *Q. variabilis* exhibit no dormancy and germinate rapidly after seed fall, generating robust taproots penetrating deep in the soil. Moreover, the robust taproots alone, even without support of cotyledon reserves, show amazing capability to regenerate into normal seedlings (Xiang et al., 2019). Therefore, we predict that the germination schedule and rapid colonization of taproots are highly responsible for the thin-tailed and right-skewed shape of the *Q. variabilis* seedling distribution than in those species exhibiting fat-tailed distributions.

We found that ^15^N enrichment in seeds can be achieved regardless of tree size of *Q. variabilis*. This implies that trees entering their flowering stage can successfully transport and distribute nitrogen isotope to their seeds via xylem systems. The high efficiency of transport and incorporation of ^15^N into seeds via xylem is well beyond our current knowledge (Horwath et al., 1992; Proe et al., 2000; Nair et al., 2014). We also noticed that ^15^N enrichment in seeds was dependent on the tree size under the same dosage of ^15^N solution. However, the tree size-dependent feature of isotope enrichment in seeds may allow for a comparison of response variables from several enriched focal trees that scattered in one landscape (Carlo et al., 2009). Unfortunately, we failed to explore the relation between ^15^N enrichment in seeds and tree size due to small sample size in this study, though it could be an interesting point in future work. The application of this technique using stable nitrogen isotope is making possible the exact identification of within-population seedlings established at long distances. Because ^15^N was successfully allocated into the mature seeds, we suggest that xylem ^15^N injection could be applied to a variety of tree species bearing large-sized seeds for tracking seed dispersal and seedling recruitment in different forest ecosystems. Therefore, our experiments with these plant species demonstrate that xylem ^15^N injection method can be a valuable tool for studies of seed dispersal and recruitment.

Swanston and Myrold (1998) found that both ^15^ NO${}_{3}^{+}$ and ^15^ NH${}_{4}^{+}$ at levels approaching 1% of crown N were effective in labeling trees. Horwath et al. (1992) reported that adding greater than 5–10% of the crown N through stem injection resulted in toxic effects on trees. In our study, however, only 2.7 g N was injected into each tree, much less than 1% of the crown N. Our pilot study has shown that there is a positive relationship between seed δ^15^N and injected ^15^N. Due to the fact that ^15^N will be diluted with the development of seedlings (Carlo et al., 2009; Yi et al., 2014), we strongly recommend xylem injection of 0.2–0.3 g ^15^N to a single tree with DBH > 10 cm for future studies, which will ensure higher levels of ^15^N enrichment in both seeds and seedlings and benefit seedling tracking in the field. Most vascular plants acquire the main available forms of nitrogen (N) as NH${}_{4}^{+}$ or NO${}_{3}^{-}$ by taking up through root absorption and then undergo xylem transport within plant parts (Oh et al., 2008); therefore, ^15^NH_4_^15^NO_3_ can be an alternative compound for xylem injection (Nair et al., 2014).

Seed manipulation of previous tracking methods has been found to cause seed death, fungal contamination, or even low germination rate (Den Ouden et al., 2005; Iida, 2006; Canner and Spence, 2011; Hirsch et al., 2012a,b; Suselbeek et al., 2013; Yi and Wang, 2015), which makes it a little bit difficult to track the real patterns of seedling recruitment in the field. However, our xylem ^15^N injection method caused no such problems because xylem injection did not involve any surgical alterations to ^15^N enriched seeds that resembled the natural counterparts, which allows tracking for actual seed dispersal kernel. Previous investigations have shown that seed manipulation by marking also affects seed consumption and predation by seed-eating animals (Alverson and Díaz, 1989; Winn, 1989; Den Ouden et al., 2005; Pons and Pausas, 2007; Yi and Zhang, 2008; Yi et al., 2011). On the contrary, our *in vivo* xylem ^15^N injection showed no significant effects on seed selection by seed-eating animals as well as seed germination and seedling development. Although the N and protein contents of acorns were slightly enhanced by xylem ^15^N injection, no significant changes were detected in seed hoarding and pilfering of *L. edwardsi*, suggesting that xylem ^15^N injection is unable to cause detectable changes in seed odor and palatability of *Q. variabilis* and then to affect actual patterns of seed dispersal. We are unable to guarantee that seed selection in the enclosures matches the seed dispersal patterns in the field; however, data collected from the enclosures usually reflect the true patterns (Xiao et al., 2008). Injecting isotopic signals into seeds through xylem appear to be an undetectable clue to seed-eating animals and have no negative effects on seed germination and seedling development. Despite lacking experimental evidence, it is not possible that xylem injection of 2.7 g N into a tree with DBH of 20–30 cm will cause significant variations of seed production. Making this judgment is based on the fact that a supplement of 15 g m^–2^ year^–1^ of nitrogen shows reliable sign of increase in acorn production (Bogdziewicz et al., 2017). Even if N xylem injection will potentially increase seed production, high level of seed availability has been shown to promote seed dispersal (Vander Wall, 2002; Jansen et al., 2004). Perhaps the main benefit of the xylem injection method is that it labels the seeds without directly affecting them. Moreover, our xylem ^15^N injection represents a passive injection system that not only avoids multiple administration of nitrogen solution onto the plant parts (Carlo et al., 2009; Putz et al., 2011; Castellano and Gorchov, 2013; Forster and Herrmann, 2014), but also benefits ^15^N enrichment in seeds along with their development. In addition, we found that drilling small shallow holes in the tree trunks for xylem ^15^N injection was unlikely to cause severe damage to trees that has already entered into their flowering stages. Therefore, the xylem ^15^N injection method is expected to be powerful to track seed dispersal and seedling recruitment mediated by hoarding animals.

Similar to the ^15^N-urea foliar-spraying methods and seed-soaking approaches (Carlo et al., 2009; Castellano and Gorchov, 2013; Forster and Herrmann, 2014; Yi et al., 2014; Yi and Wang, 2015), the xylem ^15^N injection method requires much effort to locate potential seedlings over a wide range after seeds are dispersed. However, different from other seed-labeling approaches (Wang et al., 2013), our xylem ^15^N injection method appears to be labor-saving as the multiple-step seed movements by animals were ignored. This deployment can be well accepted because seedling recruitment is regarded as the ultimate outcome of seed dispersal ecology, and most seed shadows have been estimated from spatial patterns of seedlings (Vander Wall, 1990). Some may doubt that xylem ^15^N injection will not be applicable for plants bearing small seeds (Longland and Clements, 1995). Recent studies have verified that isotopic signals can be transferred from ^15^N-enriched seeds to their seedlings for small-seeded or wind-dispersed tree species (Castellano and Gorchov, 2013; Forster and Herrmann, 2014; Wang et al., 2016). Moreover, we can collect seedlings for analysis at early stages to compensate for isotope dilution in samples (Yi et al., 2014), which facilitates us to locate and identify the focal seedlings. Moreover, our xylem ^15^N injection method permits seed removal directly from the canopies either by wind or by avian frugivores participating in long-distance dispersal, which has been verified by Carlo et al. (2009). Although tracking long-distance dispersal using this method appears to be hard, previous seed tracking methods also face this problem (Hirsch et al., 2012a). Despite this, we believe that our xylem ^15^N injection method could broaden the study of dispersal ecology and may provide a powerful tool to estimate long-distance seed dispersal. Our xylem ^15^N injection method also allows tracking seedling recruitment from many individuals within the same site by deliberately altering the ^15^N dosage levels (Carlo et al., 2009; Yi et al., 2014) or selecting trees with different DBH or crown size.

We found that acorns of *Q. variabilis* were successfully ^15^N enriched in this study, and seed-eating animals showed no significant caching and pilfering preference, implying that our xylem ^15^N injection method can be applied to a variety of tree species. Our previous ^15^N injection attempts on *Melia azedarach*, *Elaeocarpus decipiens*, *Juglans mandshurica*, *Q. mongolica*, *Q. fabri*, and *Q. variabilis* from different genera have successfully resulted in enriched ^15^N in their seeds or nuts, though low ^15^N abundance in seeds was obtained (Supplementary Figure 2), which is mainly due to the low concentration of ^15^NH_4_Cl and K^15^NO_3_ (40 mmol/L) administrated. Therefore, our xylem ^15^N injection method makes it possible to track seedling recruitment of plants regardless of seed size, which will play an important role in the seed dispersal ecology.

## Conclusion and Management Implications

In summary, we showed that xylem injection of ^15^N successfully enriched ^15^N in both the acorns and seedlings of *Q. variabilis*. We also successfully tracked seedlings established from acorns dispersed by seed-eating animals in the field ^15^N stable isotopes, verifying the validity of the ^15^N xylem injection method to track seedling establishment. We therefore suggest that xylem ^15^N injection, combined with foliar application of ^15^N, is a reliable, labor-saving and user-friendly isotopic method for seed dispersal biology. We believe that our xylem ^15^N injection method will provide new perspectives and some novel approaches to tracking seed dispersal and seedling recruitment mediated by seed-eating animals in seed dispersal ecology. Given that seed dispersal mediated by seed-eating animals and natural regeneration have been widely recognized as key processes within forest ecosystems, our tracking method using ^15^N xylem injection will be promising in better understanding the actual patterns of seed dispersal and seedling recruitment, and natural succession of forest ecosystems. In addition, the ^15^N tracking method is powerful to demonstrate the actual pattern of spatial dispersal and therefore to deepen our understanding of seed dispersal ecology, highlighting the importance of seed dispersal ecology for natural regeneration and forest management practices.

## Data Availability Statement

The datasets presented in this study are publicly available. This data can be found here: Figshare, doi: 10.6084/m9.figshare.14390918.

## Ethics Statement

The animal study was reviewed and approved by the Ethics Committee of Qufu Normal University.

## Author Contributions

XY designed the study. SY, MW, and MJ carried out the experiments. SY performed the data analyses. XY and SY wrote the first draft of the manuscript. All authors contributed substantially to revisions.

## Conflict of Interest

The authors declare that the research was conducted in the absence of any commercial or financial relationships that could be construed as a potential conflict of interest.

## Supplementary Material

Supplementary Figure 1 A map with the study area.

Supplementary Figure 2 An illustration of xylem injection of ^15^NH_4_Cl and K^15^NO_3_ into *Quercus variabilis* in the field.

Supplementary Figure 3 Isotopic values of seeds of several tree species xylem injected with 40 mmol/L ^15^NH_4_Cl and K^15^NO_3_ compared to the control.

Supplementary Figure 4 Difference in δ^15^N of enriched and natural seedlings of *Quercus variabilis* collected in the field.
