# Supplementary material for: Tracking Animal-Dispersed Seedlings Using 15N Xylem Injection Method
Source: Front Plant Sci. 2021 Apr 30;12:582530. doi: 10.3389/fpls.2021.582530 (PMC8120291; doi:10.3389/fpls.2021.582530)
Supplement: Supplementary Figure 1 — A map with the study area. [file Data_Sheet_1.docx]

**Fig. S1.**A map with the study area.

**Fig.S2.** An illustration of xylem injection of ^15^NH_4_Cl and K^15^NO_3_into *Quercus variabilis*in the field.


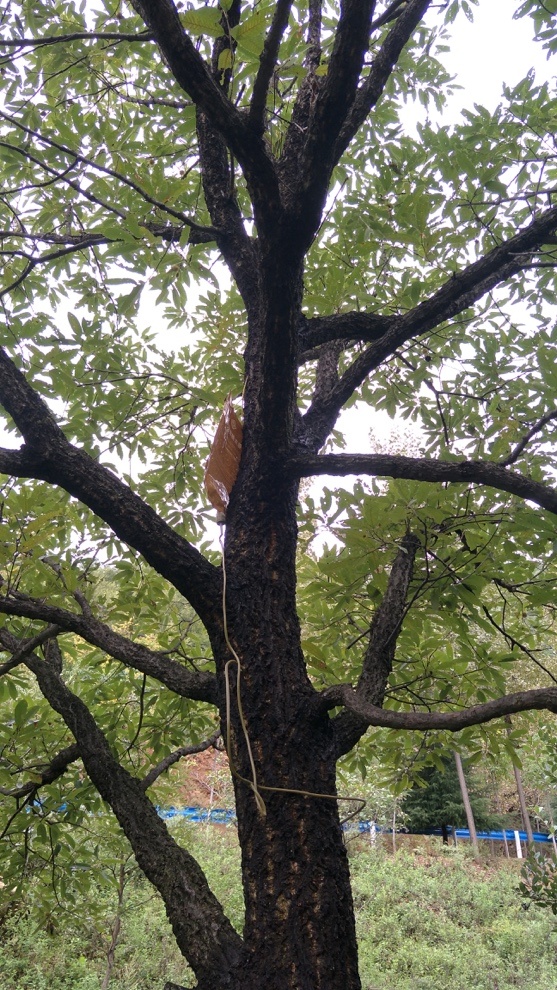


Fig. S3. Isotopic values of seeds of several tree species xylem injected with 40 mmol/L ^15^NH_4_Cl and K^15^NO_3_ compared to the control.

Fig. S4. Difference in δ^15^N of enriched and natural seedlings of *Quercus variabilis*collected in the field.
